# More advanced Alzheimer's disease may be associated with a decrease in cerebrospinal fluid pressure

**DOI:** 10.1186/1743-8454-6-14

**Published:** 2009-11-16

**Authors:** Peter Wostyn, Kurt Audenaert, Peter Paul De Deyn

**Affiliations:** 1 Department of Psychiatry, PC Sint-Amandus, Reigerlostraat 10, 8730 Beernem, Belgium; 2 Department of Psychiatry, Ghent University Hospital, De Pintelaan 185, 9000 Ghent, Belgium; 3 Department of Neurology and Memory Clinic, Middelheim General Hospital (ZNA), Lindendreef 1, 2020 Antwerp, Belgium; 4 Laboratory of Neurochemistry and Behavior, Institute Born-Bunge, University of Antwerp, Universiteitsplein 1, 2610 Antwerp, Belgium

## Abstract

In a recent article, elevated cerebrospinal fluid pressure (CSFP) consistent with very early normal pressure hydrocephalus (NPH), was found in a small subset of Alzheimer's disease (AD) patients (possible AD-NPH hybrids) enrolled in a clinical trial for chronic low-flow cerebrospinal fluid drainage. Also in the same study, was another interesting finding that merits further discussion: a substantial proportion of AD patients had very low CSFP. Based on the characteristics of these subjects, we hypothesize that more advanced AD may be associated with a decrease in CSFP. Reduced CSFP among a group of AD patients could provide a clue towards a better understanding of the high rate of comorbidity reported between AD and glaucoma since it has been shown that mean CSFP is lower in subjects with primary open-angle glaucoma. This could result in an abnormally high trans-lamina cribrosa pressure difference and lead to glaucomatous damage.

## Introduction

In a recent article published in *Cerebrospinal Fluid Research*, Silverberg *et al*. reported elevated cerebrospinal fluid pressure (CSFP), consistent with very early normal pressure hydrocephalus (NPH), in a small subset of Alzheimer's disease (AD) patients enrolled in a clinical trial of chronic low-flow cerebrospinal fluid (CSF) drainage [1]. This AD-elevated CSFP group provided further support for the existence of an AD-NPH hybrid and CSF circulatory failure. However, the same study reported another interesting finding that we believe merits further discussion, in that a substantial proportion of AD patients had very low CSFP. In the present article, we briefly summarize supportive evidence from these observations that suggest that more advanced AD may be associated with a decrease in CSFP. Interestingly, the finding of a high occurrence of reduced CSFP among patients with AD could shed new light on the link between AD and glaucoma [2,3].

## Discussion

**Figure 1:**
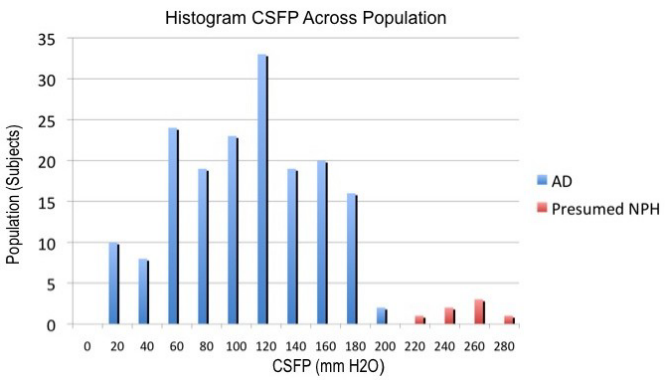
**Frequency histogram showing the distribution of cerebrospinal fluid pressure (CSFP) in all subjects with Alzheimer's disease (AD)**. Reproduced from Silverberg *et al*. [1] with permission.

An article entitled "Elevated cerebrospinal fluid pressure in patients with Alzheimer's disease", reported on CSFP in patients with AD [1]. This was a clinical trial to evaluate the safety and efficacy of low-flow shunting in subjects that met strict National Institutes of Neurological and Communicative Diseases and Stroke-Alzheimer's Disease and Related Disorders Association criteria for probable AD. The therapeutic objective guiding low-flow ventriculoperitoneal shunting as a treatment for AD was to improve safely the CSF turnover and clearance of metabolic by-products, such as β-amyloid and tau, from the brain. Subjects were carefully screened to exclude those with clinical, radiographic, or CSFP signs of NPH. As a final exclusion prior to shunt implantation, CSFP was measured supine under general anesthesia via the implanted ventricular catheter [1]. Normally, CSFP ranges from 5 - 15 mmHg (or 68 - 204 mmH_2_O) and age is not known to affect CSFP in adults [4]. During the initial implantation procedure, seven of the 181 subjects (3.9%) with no clinical or radiographic signs of NPH had an opening CSFP >200 mmH_2_O [1]. These subjects were withdrawn from the remainder of the study, because of probable associated early NPH. For this AD-elevated CSFP group, the mean CSFP was 249 ± 20 mmH_2_O. AD patients with elevated CSFP were significantly younger (67 ± 6 years vs. 74 ± 6) and significantly less demented on the Mattis Dementia Rating Scale (MDRS) (118 ± 6 vs. 106 ± 17) than those without elevated CSFP [1]. The AD group without elevated CSFP consisted of 174 subjects (the remaining 96.1%). Mean opening CSFP in this group was 103 ± 47 mmH_2_O, which was statistically significantly lower when compared to the AD-elevated CSFP group and a somewhat younger non-demented control group of subjects with Parkinson's disease (140 ± 60 mmH_2_O) [1,5]. The distribution of CSFP in all AD subjects is shown in Figure 1. Remarkably, the frequency histogram of the CSFP distribution shows a substantial proportion of AD patients with very low CSFP. Indeed, it could be argued that there were two subgroups of AD patients within the group without elevated CSFP: those with nearly normal CSFP and those with much lower CSFP (G. Silverberg, personal communication, 2008). Forty-two of the 174 subjects (24.1%) had a CSFP lower than the normal range [1]. This is a much higher proportion than one would expect from a normal population. An unexpected finding of this study was the relatively high (>30%) proportion of subjects with moderate to severe dementia as measured by MDRS total scores below 100, despite inclusion-exclusion criteria designed to capture subjects with mild to moderate dementia (Mini-Mental State Examination score between 15 and 24, inclusive) [1,6]. Although not specifically investigated in this study, cerebral atrophy associated with moderate to severe AD could be hypothesised to be associated with lower CSFP (G. Silverberg, personal communication, 2008). In the present article, we focus on the AD group without elevated CSFP. To the best of our knowledge, there is no previous study reporting a high occurrence of very low CSFP among patients with AD. Based on the characteristics (older and more demented on the MDRS) of the AD patients without elevated CSFP, we hypothesize that more advanced AD may be associated with a decrease in CSFP.

The finding of a high occurrence of reduced CSFP among AD patients is of major importance because it may provide a better understanding of the high rate of comorbidity reported between AD and glaucoma [2,3]. In a nursing home-based study in Germany, the prevalences of glaucoma (as defined by characteristic optic nerve or visual field changes) were reported to be 25.9% in patients with AD and 5.2% in controls [3]. In another study, Tamura *et al*. found that the prevalence of open-angle glaucoma (OAG) in Japanese patients with AD was 23.8%, which was significantly higher than that of the control subjects (9.9%) [2]. There was no significant difference between intraocular pressures (IOPs) in AD patients with OAG and without OAG, and almost all AD patients with OAG showed normal tension. Furthermore, in a retrospective case-control study, Berdahl *et al*. reported the intriguing new observation that mean CSFP was 33% lower in subjects with primary open-angle glaucoma (POAG) (9.2 mmHg) compared with that of nonglaucomatous controls (13.0 mmHg; *P *< 0.00005) [4]. The authors noted that their observation supports the concept that an abnormally high trans-lamina cribrosa pressure difference plays an important role in glaucomatous optic nerve damage, whether the result of elevated IOP, reduced CSFP or both. Considering the above data, it seems reasonable to speculate that there may be a causal relationship between AD and glaucoma that may be explained by low CSFP in patients with AD. This obviously needs to be confirmed by future research.

It should be stressed that other factors might also explain the link between AD and glaucoma. For example, Tamura *et al*. evaluated the APOE ε4 allele as a common genetic risk factor for AD and OAG [2]. However, the percentage of AD patients with OAG who carried an APOE ε4 allele was not significantly different to that of AD patients without OAG, suggesting that APOE ε4 polymorphism may not be a common risk factor predisposing to both disorders [2]. Recently, Helicobacter pylori infection has also been suggested to be involved in the pathogenesis of both AD and glaucoma [7]. To investigate whether primary open-angle glaucoma is associated with increased risk of developing AD, Kessing *et al*. carried out a nationwide case register study comparing the rate of subsequent AD for patients with POAG (including normal tension glaucoma) with the rate for patients with primary angle-closure glaucoma (PACG), cataract, and osteoarthritis (OA) and with the rate for the general population [8]. All patients included in the study were identified at hospital admission or outpatient contact during the period 1977 to 2001 in Denmark. However, the hypothesis that patients with POAG have increased risk of developing AD was not supported by the results [8].

## Conclusion

In conclusion, we have summarized supportive evidence from the observations of Silverberg *et al*. that suggest that more advanced AD may be associated with a decrease in CSFP. The finding of a 24.1% occurrence of reduced CSFP among 174 patients with AD could be linked to the high rate of comorbidity between AD and glaucoma, since it has been shown that mean CSFP is lower in subjects with POAG. This could result in an abnormally high trans-lamina cribrosa pressure difference and lead to glaucomatous damage.

## Competing interests

The authors declare that they have no competing interests.

## Authors' contributions

All authors contributed equally to this work. All authors read and approved the final manuscript.
